# Concomitant Severe Psoriasis and Bullous Pemphigoid Induced by COVID-19

**DOI:** 10.3390/tropicalmed8020107

**Published:** 2023-02-08

**Authors:** Fabrizio Martora, Teresa Battista, Gabriella Fabbrocini, Matteo Megna

**Affiliations:** Dermatology Unit, Department of Clinical Medicine and Surgery, University of Naples Federico II, 80131 Naples, Italy

**Keywords:** COVID-19, bullous pemphigoid, psoriasis, chronic inflammatory disease, skin manifestations, pandemic, herpes zoster, pemphigus, atopic dermatitis, vaccine

## Abstract

Coronavirus disease 2019 (COVID-19), caused by severe acute respiratory syndrome coronavirus 2 (SARS-CoV-2), first isolated in Wuhan, China, is currently a pandemic. At the beginning of the pandemic, pulmonary issues were the most discussed and studied. However, now 3 years later, the role of the dermatologist has become increasingly central. Often the diversity in the presentation of these manifestations has made it difficult for the dermatologist to recognize them. In addition to the common symptoms involving fever, cough, dyspnea, and hypogeusia/hyposmia that have been widely discussed in the literature, much attention has been paid to dermatologic manifestations in the past year. The vaccination campaign has been the most important strategy to combat the COVID-19 pandemic. Specifically, two viral vector-based vaccines [Vaxzervria^®^ (AstraZeneca; AZD1222) and COVID-19 Janssen^®^ vaccine (Johnson & Johnson; Ad26.COV2. S)] and two mRNA-based vaccines [Comirnaty^®^ (Pfizer/BioNTech; BNT162b2) and Spikevax^®^ (Moderna; mRNA-1273)]. However, several cutaneous adverse reactions have been reported following vaccination, making the dermatologist’s role critical. It is possible to group these adverse reactions according to a classification with six main clinical pictures: urticarial rash, erythematous/maculopapular/morbid rash, papulovesicular rash, chilblain-like acral pattern, livedo reticularis/racemose-like, and purpuric “vasculitic” pattern. Beyond this classification, there are several reports of other dermatologic manifestations associated with the infection, such as pityriasis rosea, herpes zoster, or, particularly, the worsening of pre-existing chronic inflammatory dermatologic diseases. Here we report the case of a 61-year-old patient who presented at our clinic with a diffuse psoriasiform eruption mixed with a concomitant blistering rash induced by COVID-19. The uniqueness of our case has two features: the first is the concomitance of the two events after infection that seems to be unprecedented; the second is the management of the patient that could help dermatology colleagues in the management of these conditions during infection.

## 1. Introduction

The skin manifestations associated with SARS-CoV-2 infection are numerous and heterogeneous. The knowledge of skin manifestations at the beginning of the pandemic was very limited; to date, we have managed through the many reports in the literature to produce a classification of these manifestations. We can subdivide the manifestations into six patterns involving inflammatory reactions (maculopapular/morbilliform, urticarial, and vesicular eruptions) or vascular (chilblain-like eruptions, petechiae/purpuras, and livedo racemose) [1,2]. The dermatologic findings are secondary to the binding of SARS-CoV-2 to angiotensin-converting enzyme-2 (ACE2) receptors present in cutaneous blood vessels, eccrine gland epithelial cells, and the basal layer of hair follicles [2]. Two online registries have been created by the American Academy of Dermatology (AAD) and International League of Dermatological Societies (ILDS) where the reaction associated with the infection can be recorded; this has to date allowed for a much broader knowledge of these manifestations [3]. In addition to these widely described patterns, there are many reports in the literature where COVID-19 infection is associated with other dermatologic diseases such as pityriasis rosea, herpes zoster, lichen planus, or even a worsening of chronic inflammatory diseases such as psoriasis, pemphigus, atopic dermatitis associated during COVID-19 infection [4,5,6]. Another interesting finding is that these manifestations often arise before the onset of respiratory disease or several days after the infection. Finally, they can be found in paucisymptomatic patients.

## 2. Detailed Case Description

**Figure 1:**
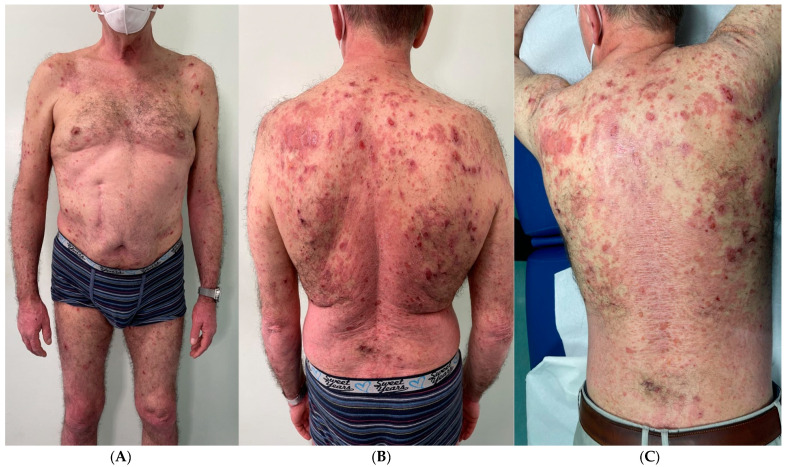
(**A**–**C**) Severe psoriasis worsening was experienced, with plaques involving the entire dorsal region and extending to the upper and lower limbs (PASI 15.2 and BSA 24%). Numerous bulla appeared at the scapular and clavicular regions, as well as at the upper and lower limbs.

Here we report the case of a 61-year-old patient who presented at our clinic with a diffuse psoriasiform eruption mixed with a concomitant blistering rash suspected to be induced by COVID-19 (Figure 1). The patient’s medical history was negative except for the presence of mild psoriasis for 10 years, localized mainly to the lumbar region and elbows, which was well controlled with topical calcipotriol and betamethasone. He was taking no other medications. Prior to the visit to our hospital, the patient reported that he contracted COVID-19 (D0). The infection lasted 10 days asymptomatically and without taking any medication. A molecular swab on day 10 was negative (D10). He presented the negative swab seven days after (D17) with a severe worsening of psoriasis, with plaques that involved the entire dorsal region and extended to the upper and lower extremities (PASI 15.2 and BSA 24%). The patient had been vaccinated against COVID-19 infection with three doses of the Moderna mRNA-1273 vaccine. The vaccination had been completed about three months before the COVID-19 infection described.

After another three days, then 10 days after the swab negativization and 20 days after the COVID-19 infection (D20), numerous bullae appeared in the scapular and clavicular regions as well as in the upper and lower limbs.

**Figure 2:**
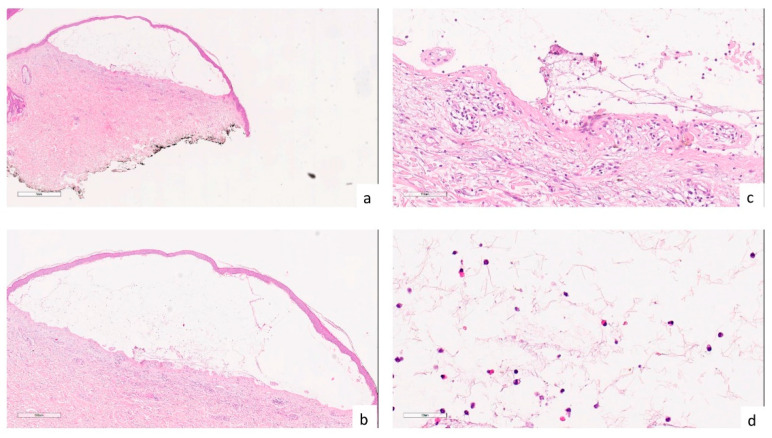
(**a**,**b**) Histologically, a subepidermal bullous lesion is observed ((**a**) hematoxylin-eosin, 2×; (**b**) hematoxylin-eosin, 4×); (**c**) Drawing of the dermal papillae on the bulla floor (hematoxylin-eosin, 20×); (**d**) at higher magnification, the bulla content consisting of fibrin and a modest cellular component, including an appreciable proportion of eosinophils, is apparent (hematoxylin-eosin, 40×).

A skin biopsy showed a subepidermal bullous lesion whose contents were composed of an appreciable proportion of eosinophils (Figure 2). The patient was tested for BP 180 and BP 230 autoantibodies, which were positive. Direct immunofluorescence confirmed biopsy results and autoantibody positivity. Hence a diagnosis of suspected COVID-19-induced psoriasis exacerbation and concomitant bullous pemphigoid (BP) was made. Acitretin 35 mg daily and azathioprine 100 mg daily led to a significant improvement in both diseases in 10 weeks [7].

The treatment was well tolerated, and no side effects were reported. After 10 weeks of treatment, we began a slow and gradual reduction of ongoing therapy.

## 3. Discussion

Psoriasis is a chronic inflammatory skin disease characterized by the appearance of scaly, indurated, and erythematous plaques [8,9]. On histology, three main histologic features can be appreciated: epidermal hyperplasia, dilated and prominent blood vessels in the dermis, and an inflammatory infiltrate of leukocytes, predominantly in the dermis [9].

The skin is not the only part that can be involved; psoriasis can often affect both joints and nails. Recently, the concept of psoriatic march has been introduced as psoriasis can be associated with several comorbidities such as obesity, metabolic syndrome, diabetes, and hypertension, as well as having a strong impact on the patient’s quality of life [8]. The pathogenesis of psoriasis involves antimicrobial peptides (AMPs), dendritic cells (DCs), tumor necrosis factor (TNF)α, interleukin (IL)23, Th17, IL17, IL22, and signal transduction and activation transcription (STAT) [7]. All these characteristics influence the choice of treatment for the disease [10]. Available therapies vary depending on the patient’s clinical picture; local treatments remain the first-line therapy for mild psoriases, such as topical corticosteroids, keratolytics, vitamin D3 derivatives, and calcineurin inhibitors [8]. Phototherapy is directed at treating the more extensive forms; often, its use in combination with other therapies leads to excellent therapeutic results [8,9,10]. For moderate-severe forms, in addition to the classic conventional drugs such as acitretin, methotrexate, and cyclosporine, today we have multiple therapies available thanks to the advent of apremilast Jak inhibitors or biologic drugs that have made the treatment of severe forms easier for the dermatologist [9,10,11].

Bullous pemphigoid is the most frequent of the autoimmune bullous diseases; it is found in about 70% of autoimmune diseases [12]. It predominantly involves elderly subjects, although many occurrences have also been reported in adults or children [10]. The rash is characterized by taut, often large blisters. Itching is generally very intense. The localizations prefer the flexural surface of the limbs, the anteromedial surface of the thighs, and the abdomen. The oral mucosa is affected in 10% of cases. Bullous pemphigoid is an autoimmune disease caused by antibodies directed against two molecules located in the hemidesmosomes of keratinocytes of the basal layer of the epidermis: BP 180 and BP 230 proteins [13]. Skin biopsy of the bulla is essential; on histology, a subepidermal dislocation is found; the bulla contains fibrin and cellular elements such as neutrophilic granulocytes and eosinophils. Through immunofluorescence examinations, the presence of linear IgG and/or C3 deposits along the dermal-epidermal junction can be noted [12,13,14]. The choice of treatment varies according to the patient’s comorbidities since most who are affected are elderly; localized forms should be treated with low-potency topical corticosteroids, while extensive forms should be started with oral corticosteroid therapy 1 mg/kg/day for long periods to avoid recurrence. Immunosuppressants such as azathioprine, chlorambucil, and methotrexate are to be used as second-line treatment. Third-line treatment, on the other hand, involves intravenous immunoglobulin administration of rituximab (anti-CD20 Ab) or an anti-IgE antibody (omalizumab) [12,13,14].

The coexistence of psoriasis with autoimmune bullous diseases (AIBDs), particularly bullous pemphigoid (BP), has been documented in case reports and series, as well as in epidemiological studies.

Usually, the onset of psoriatic manifestations precedes that of BP. The clinical picture would be toward younger patients with a less severe picture than a classic patient presenting with isolated BP. Another interesting finding from recent studies on the coexistence of these pathologies is that they seem to favor the female sex as opposed to isolated BP cases where the male sex is more generally affected.

The underlying pathogenetic mechanisms have not yet been demonstrated; various hypotheses are being investigated. The most interesting one at present is that some cases of coexistence of these two diseases can be identified as an anti-laminin gamma-1 pemphigoid, a rare form that has recently been recognized as a distinct entity and may mimic BP and/or other subepidermal AIBDs [15].

The epitope-spreading phenomenon described in the literature predicts the following explanation: injury secondary to an inflammatory process such as psoriasis can cause exposure to antigens that at the same time can cause the onset of autoimmune bullous diseases such as BP.

This would seem to be the most widely accepted hypothesis correlating the two diseases.

To date, several reports on the potential role of COVID-19 in the worsening of psoriasis are available [16]. Recently, Aram et al. [17] published a review of the published literature where they discussed the relationship between COVID-19 and the exacerbation of some dermatological diseases; regarding psoriasis, the authors found nine cases of exacerbation of the disease during COVID-19 infection in the literature; many data regarding the therapy taken during these relapses are missing [17].

Concerning the new onset of bullous pemphigoid with COVID-19, to the best of our knowledge, a case in the literature was described by Olson et al. [18]. The authors report that the rash appeared 3 days before the patient tested positive for infection, but the history is unclear as the patient was obese and diabetic and was using medications which, as reported in the literature, could be the cause of the onset of these manifestations [18].

Goon et al. [19] described a case of an 82-year-old patient who contracted a COVID-19 infection that lasted 6 weeks; the patient first developed a diffuse rash and then, in the fourth week, bullae appeared, diagnosed as BP on biopsy [19].

Additionally, there are several reports of de novo or exacerbation of this condition following COVID-19 vaccination [20].

The reason could be the release of inflammatory cytokines such as interleukin (IL)-1B, IL-6, IL-8, and IL17, which are the main actors in the pathogenesis of both psoriasis and the immunological reactions induced by SARS-CoV-2 [21]. This may only partly explain the occurrence of these two events and, at the same time, be able to correlate them; several large-scale studies will be needed to confirm this, which remains a hypothesis.

There are very few reports in the literature correlating psoriasis and BP with COVID-19, as we discussed [18,19], unlike COVID-19 vaccination for which several reports have been identified as a potential cause of worsening or onset of BP or for other dermatological conditions [22,23,24,25,26,27].

Several reviews or meta-analysis studies conducted on a large scale are available in the literature, where all the information gathered to date on the topic is pooled [28,29,30]. A solid underlying mechanism that can correlate COVID-19 infection or vaccination with skin manifestations has not yet been found, and more research on the topic is needed.

To the best of our knowledge, this is the first case of concomitant COVID-19-induced psoriasis worsening and new BP onset. We believe that the management we have carried out for these two events concerning COVID-19 infection can be very useful literature for the future.

## 4. Conclusions

In conclusion, we believe that almost three years after the outbreak of the pandemic, there is still scant scientific evidence that would explain how skin manifestations may develop after COVID-19 infection; although the rate of new COVID-19 cases is lower than in the past, new possible correlations between viral infection and cutaneous diseases continue to arise. Given the greater amount of current knowledge about the virus and its activity, this should encourage researchers to conduct new studies to deepen the awareness of new possible associations between autoimmune skin diseases and SARS-CoV-2. The role of the dermatologist is, therefore, becoming fundamental in two aspects: the first is in recognizing these manifestations early, and the second is in managing the treatment during the COVID-19 infection. In the future, suitable guidelines and standardized classifications will be needed in order to be able to act in the best possible way. Great cooperation between all dermatologists would be desirable in order to be able to draw up updated classifications and treatment guidelines together. In recent years we have focused more on the explanation of the event than on the treatment of these reactions; our case report highlights a new particular reaction involving two different events during the infection, and at the same time, it also provided a cure that can be of assistance to future readers. We hope that after our case, new studies can be conducted with a wide range of patients by other dermatologist colleagues in order to have more scientific evidence. Finally, we hope that new registers will be set up or those previously made will be updated in order to have an ever-increasing number of cases in the literature. Certainly, further studies are still needed today.

## Data Availability

Data are reported in the current study and are available on request from the corresponding author. Data sharing is not applicable to this article as no datasets were generated or analyzed during the current study.
